# Is There Any Correlation between Insulin Resistance and Nitrate Plasma Concentration in White Coat Hypertensive Patients?

**DOI:** 10.4061/2009/376735

**Published:** 2009-08-24

**Authors:** Leila Maria Marchi-Alves, Evelin Capellari Carnio

**Affiliations:** Departamento de Enfermagem Geral e Especializada, Escola de Enfermagem de Ribeirão Preto/Universidade de São Paulo, Avenida Bandeirantes, 3900, 14040-902-Ribeirão Preto, SP, Brazil

## Abstract

We evaluated a relationship between nitric oxide plasma correlation and insulin resistance in white coat hypertensive patients. Patients were screened for white coat hypertension using an ambulatory blood pressure monitoring. The homeostasis model assessment insulin resistance index (HOMA-IR) was used to assess insulin resistance, and plasma nitrate concentrations were determined by chemiluminescence. The HOMA-IR was significantly higher in hypertensive (3.84 ± 0.62) when compared to white coat (2.11 ± 0.36) and normotensive (1.40 ± 0.21). We found no correlation between HOMA-IR and plasma nitrate levels in all three groups. We suggest that the more important issue for these patients is to focus on the metabolic abnormalities where lifestyle interventions such as weight control and exercise have proven effective.

## 1. Introduction

Insulin resistance plays a role in type 2 diabetes and is close related with public health problems, as obesity, dyslipidemias, coronary artery disease, hypertension, and a bunch of metabolic and cardiovascularmalfunction which is defined as metabolic syndrome. Insulin resistance is an alteration in the actionof insulin since besides the normal levels of insulin it cannot trigger the signal for glucose absorption. The effects of this condition can have profound pathophysiologic effects on various organs and tissues of the body. The clinical consequences, beyond hypertension, include hyperglycemia-induced tissue damage, dyslipidemia, metabolic syndrome, and cardiovascular disease [1].

Endothelium has many different roles including the regulation of blood-tissue permeability and vascular tonu, and control of vascular surface properties for during inflammation. Nitric oxide (NO) is a gaseous modulator, which acts in regulation of endothelial functions. An NO deficiency, which is also known as endothelial dysfunction, is the first step for the occurrence of many diseases including heart failure, diabetes mellitus, dyslipidemia, insulin resistance, and hypertension [2].

White coat hypertension is a well-known clinical situation defined as a persistently elevated blood pressure in the physician's office, even though the blood pressure is normal in other situations, and its prognostic significance remains controversial [3, 4]. Whether individuals with WCH have an abnormal autonomic-cardiac regulation similar to that observed in sustained hypertensive patients is unknown, and in subjects with white coat hypertension it is unclear whether ambulatory blood pressure progresses over time and exhibits an increased cardiovascular risk. Additionally, it remains unclear whether white coat hypertension is already associated with vascular end-organ damage.

Some reports have suggested that white coat hypertension might be associated with someofthese metabolic disturbances, instead of white coat hypertension per se, thatmay potentially explain the greater extent of end-organ damage sometimes observed in white coathypertension patients when compared with normotensive individuals [5].

The combination of insulin resistance and compensatory hyperinsulinemia increases the likelihood that an individual will be hypertensive. Given the rapid increase in the number of clinical syndromes and abnormalities associated with insulin resistance/hyperinsulinemia, it seems reasonable to suggest that the cluster of these changes could be related to the defect in insulin action, which can be subsumed under the term of the insulin resistance syndrome. In addition to the identification of additional clinical syndromes related to insulin resistance/hyperinsulinemia, a number of new risk factors have been recognized that would increase cardiovascular risk in these individuals. Evidence is also accumulating that sympathetic nervous system activity is increased in insulin resistant, hyperinsulinemic individuals, and, along with the salt sensitivity associated with insulin resistance/hyperinsulinemia, increases the likelihood that these individuals will develop essential hypertension [6].

The ability of insulin to stimulate glucose disposal varies more than sixfold in apparently healthy individuals [7].

Overweight and obesity are highly associated with multiple comorbidities, such as elevated blood pressure values, dyslipidaemia, reduced insulin sensitivity, and alterations of large and minor vessels. Abdominal obesity is a risk factor for cardiovascular disease worldwide, and it is becoming a dramatic issue for national health systems [8]. 

The more overweight/obese the persons are, the more likely they are to be insulin-resistant and at increased risk of cardiovascular disease, but substantial numbers of overweight/obese individuals remain insulin-sensitive, and not all insulin-resistant persons are obese. Of greater clinical relevance is evidence that the metabolic benefit and decrease in risk of cardiovascular disease following weight loss occurs primarily in those overweight/obese individuals that are also insulin-resistant [6]. 

On the other hand, cardiovascular diseases are the most common causes of deaths, particularly in developed countries, and most are related with HT. Thus, BP control is the basis for the prevention of cardiovascular death. However, the diagnosis and management of hypertension is difficult due to the fact that BP varies greatly depending on physical and mental stresses [9]. 

Insulin resistance, obesity, and endothelial dysfunction are observed in some patients suffering from hypertension. It remains unclear whether patients with white coat hypertension elude the risk of this condition; so in this study we evaluated a relationship between nitrate plasma correlation and insulin resistance in white coat hypertension patients.

## 2. Materials and Methods

### 2.1. Subjects

Based on BP measurements at the medical office and on Ambulatory Blood Pressure Monitoring (ABPM), the patients were divided into the following categories: (a) sustained hypertension—systolic BP at the medical office ≥140 mmHg or diastolic BP ≥90 mmHg, or both, and mean of systolic and diastolic BP on ABPM during the wakefulness period ≥135 mmHg and ≥85 mmHg, respectively; (b) white coat hypertension—systolic BP at the medical office ≥140 mmHg or diastolic BP ≥90 mmHg, or both, and mean of systolic and diastolic BP on ABPM during the wakefulness period <135 mmHg and <85 mmHg, respectively; (c) normotension—systolic BP at the medical office <140 mmHg and diastolic BP <90 mmHg, and mean of systolic and diastolic BP on ABPM during the wakefulness period <135 mmHg and < 85 mmHg, respectively.

Exclusion criteria for entry in the study were proceeding from the pediatric clinic, pregnant patients, insulin using diabetics, and patients with devastating illnesses including malignancies, renal failure, liver diseases, and heart failure.

The procedures followed were in accordance with the ethical standards of the responsible committee on human experimentation and with the Helsinki Declaration and were approved by the Institutional Review Committee. Subjects gave informed consent. Study population came from the same geographic area.

### 2.2. Office BP Measurements

At the medical office, BP was measured with a regularly checked automate oscillometric device (OMRON) with an appropriate cuff for the arm's circumference. After a 5-minute rest with the patient seated, the measurements were taken on the patient's bare right arm, which was supported and maintained at the heart's level. The average of three measurements was taken as the mean systolic and diastolic pressures.

### 2.3. Ambulatory BP Monitoring (ABPM)

ABPM was performed with an oscillometric device (SpaceLabs 90207), which was checked monthly against a mercury column sphygmomanometer. The measurements were taken every 15 minutes from 7:00 AM to 6:00 PM and every 30 minutes from 6:01 PM to 6:59 AM, and the patient maintained his usual activities throughout the day. The appropriate cuff for the arm's circumference was placed on the no dominant arm, and the patients were instructed to maintain their arm stretched along their body and not to move their arm during measurements. The recording valid for analysis had a minimum duration of 24 hours and 80 valid readings, corresponding to at least 80% of all measurements.

### 2.4. Anthropometric Parameters

The anthropometric indicators were analyzed according to the criteria of World Health Organization—WHO [10]. Anthropometric measurements included weight, height, body mass index (BMI), and waist circumference. Every subject's height was measured in centimeters while the participant stood still without shoes, and weight was measured to the nearest 0.1 kg with electronic weight scale in kilograms with the participant lightly clothed. Subjects were categorized according to their body mass index (BMI). BMI was calculated as weight divided by square of height (kg/m^2^). Underweight was defined as a BMI less than 18.5 kg/m^2^, normal weight as 18.5–24.9, overweight as 25–29.9 kg/m^2^, and obesity as a BMI of 30.0 kg/m^2^ or greater. Waist circumference (WC) was measured in centimeters at the midpoint between the bottom of the ribs and the top of the iliac crest. The cutoff points for cardiovascular disease risk was 102 cm for men and 88 for women.

### 2.5. Laboratory Methods

Subjects reported to the laboratory between 07:00 and 09:00 A.M., rested quietly for 10 minutes in the supine position, and a blood sample for the determination of glucose and insulin was obtained from an antecubital vein and collected into a specific tube. Blood samples were drawn after a fasting period of 12 hours at the same time of the day. Serum collected was sent to a certified medical laboratory for subsequent analysis using automated enzymatic procedures with calculated precision values <3%. An estimation of insulin resistance was calculated using the homeostasis model analysis (HOMA-IR) using the formula: glucose (mU/L) × [insulin (pmol/L)/22.5] [11].

Blood samples for nitrate determination were centrifuged immediately at 3000 g for 15 minutes, and supernatant was collected and frozen at −20°C until assayed. Since NO is stoichiometrically converted to nitrate and nitrite, the levels of the NO produced in a response can be tracked by assaying for nitrate and nitrite. The NO levels were measured after enzymatic conversion of all nitrate to nitrite. Fasting peripheral venous blood samples for nitrate were collected into heparinized vacutainer tubes. Plasma samples were isolated by 2500 g centrifugation at 4°C for 10 minutes. All plasma samples were kept at −70°C until the experiments were performed.

On the day of the assay, plasma samples were thawed and deproteinized with 95% ethanol (at 4°C) for 30 minutes, subsequently centrifuged, and the supernatant was used for measurement of nitrate according to the NO/ozone chemiluminescence technique as previously described by Archer [12], using a Sievers NO Analizer (Sievers 280 NOA; Sievers, Boulder, CO). Sodium nitrate (Sigma Chemical) was used as a standard reference.

### 2.6. Statistical Method

The values of the selected variables were defined as mean ± SD. The NO values of the groups (normotensive, hypertensive, and white coat hypertensive) were compared with one-way ANOVA (Tukey HSD). “*P*” values <.05 were considered statistically significant. *Pearson correlation analysis was used to find out the correlations between HOMA-IR and nitrate plasma concentration.*

## 3. Results

In this study 71 patients were screened for white coat hypertension, of which 64 were included in the study. The study group consisted of 20 subjects with white coat hypertension (30% male and 70% female patients), 22 hypertensives (68.2% male and 31.8% female patients), and 22 normotensive participants (31.8% male and 68.2% female patients). 

Clinical characteristics of subjects of the three groups are listed in Table 1. From the total 27.27% of normotensives, 31.82% of hypertensives and 28.57% of white coat hypertensives were overweight. The occurrence of obesity was 9.09% of normotensives, 50.62% of hypertensives, and 14.28% of white coat hypertensives.

**Figure 1:**
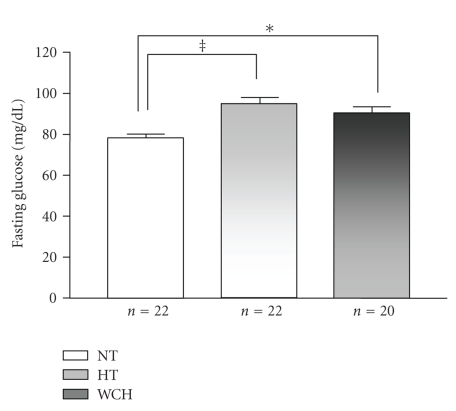
Fasting glucose levels (mg/dL) in normotensive (NT), sustained hypertensive (HT), and white coat hypertensive (WCH) patients. ^‡^ *P* < .001, *P < .05.

Plasma glucose levels of the three groups are shown in Figure 1. The HTs (95.0 ± 3.26 mg/dL) and white coat hypertensives (90.07 ± 3.72 mg/dL) had significantly higher levels of glucose than the normotensives (78.14 ± 2.05 mg/dL). No significant difference was observed between white coat hypertensives and hypertensives regarding these parameters. 

**Figure 2:**
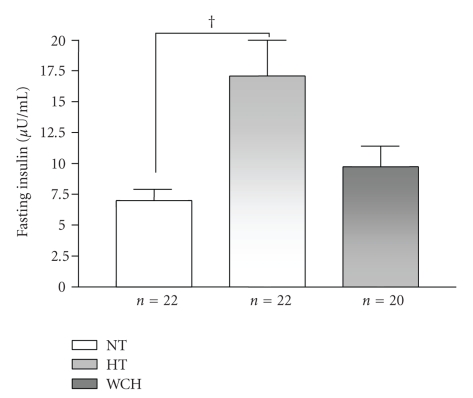
Fasting insulin levels (*μ*U/ml) in normotensive (NT), sustained hypertensive (HT), and white coat hypertensive (WCH) patients. ^†^ *P* < .01.

Figure 2 shows fasting plasma insulin concentrations in all three groups tested. There was a statistically significant difference (*P* < .01) between the hypertensive group (17.10 ± 0.91 *μ*U/mL) and the normotensive group (6.99 ± 0.91 *μ*U/mL). In addition, there was no significant difference regarding fasting plasma insulin concentrations between the white coat hypertensives (9.73 ± 1.68 *μ*U/mL) and the normotensives or hypertensives groups. 

**Figure 3:**
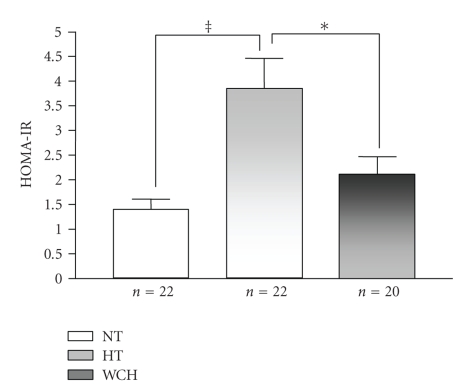
HOMA-IR in normotensive (NT), sustained hypertensive (HT), and white coat hypertensive (WCH) patients. ^‡^ *P* < .001, *P < .05.

The HOMA-IR values in the groups are demonstrated in Figure 3. The values was significantly higher in hypertensives (3.84 ± 0.62) when compared to white coat hypertensives (2.11 ± 0.36) and normotensives (1.40 ± 0.21). There were no significant differences between white coat hypertensives and normotensives. 

**Figure 4:**
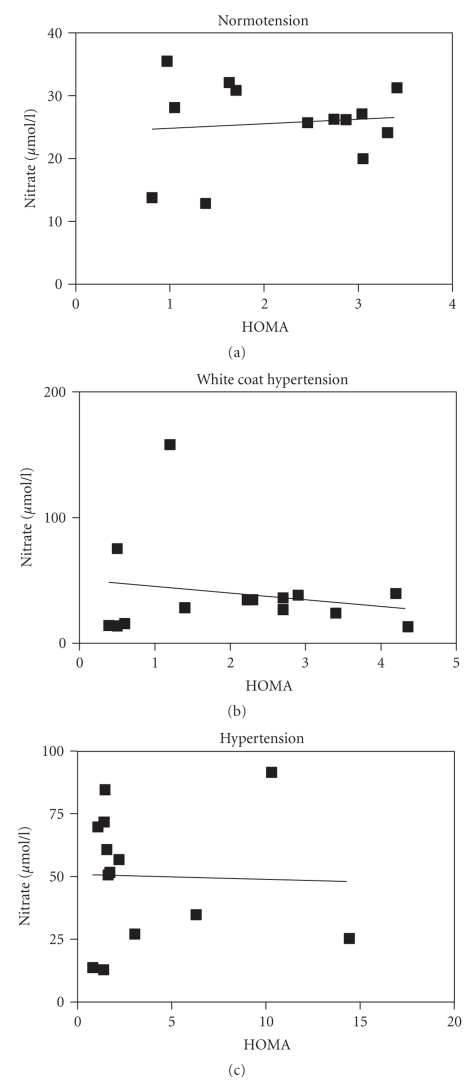
Relationship between HOMA and plasma nitrate levels in normotensive (NT), sustained hypertensive (HT), and white coat hypertensive (WCH) patients.

Figure 4 shows the correlation between HOMA-IR and nitrate plasma concentration. We found no correlation between HOMA-IR and plasma nitrate levels in all three different groups. 

## 4. Discussion

The results of this study demonstrate a normal insulin resistance in white coat hypertensive patients. 

Although the white coat hypertensive group had similar fasting insulin levels compared with normotensive group, careful inspection of their data shows that their white coat hypertension population had characteristics of the metabolic syndrome (increased body mass index and fasting glucose level), which were also present in patients with sustained hypertension. 

Although the majority of individuals in the general population that can be considered insulin resistant are also overweight/obese, not all overweight/obese persons are insulin resistant. Of greater clinical relevance is the fact that significant improvement in these metabolic abnormalities following weight loss is seen only in the subset of overweight/obese individuals that are also insulin resistant. In view of the large number of overweight/obese subjects at potential risk to be insulin resistant/hyperinsulinemic (and at increased cardiovascular risk), and the difficulty in achieving weight loss, it seems essential to identify those overweight/obese individuals who are also insulin resistant and will benefit the most from weight loss, then target this population for the most-intensive efforts to bring about weight loss [7]. 

In relation to results of HOMA IR, researchers assumed different methods to describe a threshold, such as the top quintile and 90th percentile of the HOMA in normal glucose tolerant nonobese populations. Considering the patients whose HOMA-IR is above the 90th percentile as insulin resistant subjects, Geloneze et al. [13] found a threshold value for insulin resistance of 2.71. In accordance with these criteria, inside of this proposal, our findings determine that the insulinoresistance is present in the carriers of essential hipertensive and normotensive individuals a presence of indices of normality in the evaluation of this parameter. But, in interesting way, when we evaluate the group white coat hypertension, even so let us not find statistically difference between normotension, we observe raised indices, that do not allow to fit them in the category of normoglycemics [13]. However, to validate the potential use of HOMA-IR values in clinical practice, it is important to establish its distribution in a sample of normoglycemic individuals from a population with the same genetic background [14]. In this regard, Ghiringhello et al. [15] describe the distribution of HOMA-IR values in a large sample of Brazilian population (1.2 ± 0.65 para IMC < 25, 1.8 ± 0.98 para IMC = 25–30 e 2.9 ± 1.6 para IMC > 30). Therefore, if we calculated the HOMA-IR threshold in those subjects who fulfilled this criteria, we would find that the essential hypertensive subjects as well as the white coat hypertensives present a higher index than that observed at the normal criteria,having had to be categorized as insulin-resistant. That is, if we considered this criterion of definition for insulin-resistance determination, the results point that the insulin resistance is definitivelyinstalled in white coat hypertension [15].

Although stated that the white coat hypertension group had similar fasting insulin and HOMA-IR results, compared to normotensives, careful inspection of our data shows that their white-coat hypertension population had characteristics of the insulin-resistance syndrome: elevated body mass index, fasting glucose levels, and estimated insulin resistance. Thus, we agree to previous studies that they indicate a possible relationship between end-organ damage and insulin resistance in patients with white coat hypertension exists [16, 17]. The association between white-coat hypertension and metabolic disturbances of insulin-resistance syndrome may help explain the high prevalence of cardiovascular disease often associated with white coat hypertension [18–20].

White coat hypertension and metabolic abnormalities characteristic of insulin-resistance syndrome coexist, and in which the increased risk of target organ damage is determined not by the BP, but by the metabolic abnormalities. This distinction would account for some previous findings of increased target organ damage in white coat hypertension [20].

## 5. Conclusions

White coat hypertension should preferentially be accepted as an alarming sign of excess weight and many associated disorders in the future, rather than just being considered a predisposing factor of hypertensive or atherosclerosis alone [21]. There are limitations in this study, which was conducted on a small sample of patients, and the subjects are relatively young with no inclusion criteria. Thus, although no hypertension-related target organ damage was detected in the present study, white coat hypertension cannot be regarded as a benign phenomenon because these subjects display metabolic abnormalities that are powerful risk factors for future atherosclerotic events. So in conclusion we suggest that the more important issue for the patients is to focus on the metabolic abnormalities related to the insulin-resistance syndrome where lifestyle interventions such as weight control and exercise have proven effective. However we fail to prove that it could be a possible interaction between a decreased NO bioavailability, endothelial dysfunction, and the development of insulin resistance in white coat hypertensive patients.

Arterial hypertension remains a devastating disease. All current medical therapy is expensive, complex and largely palliative. The management of hypertensive patients requires co-ordination between the patient, their support system, and healthcare professionals. Contemporary practice requires responsibility and a role in the primary prevention, detection and treatment of hypertension [22]. Healthcare professionals need to combine scientific knowledge and practice to determine the prognosis and treatment of white coat hypertension, in an effort to provide the highest quality care to the patient [23].

## Figures and Tables

**Table 1:** Clinical characteristics of normotensive (NT), hypertensive (HT), and white coat hypertensive (WCH) patients.

	NTs (*n* = 22)	HTs (*n* = 22)	WCHs (*n* = 20)
Clinic Systolic BP (mmHg)	114.9 ± 2.8	154.2 ± 3.1^‡^	142.1 ± 1.75^‡§^
Clinic Diastolic BP (mmHg)	76.1 ± 0.8	98.6 ± 2.1^‡^	90.21 ±3.4^‡§^
Ambulatory Systolic BP (mmHg)	102.9 ± 8.5	143.9 ± 3.9^‡^	118.5 ± 1.4^‡#^
Ambulatory Diastolic BP (mmHg)	71.79 ± 1.24	92.21 ± 3.0^‡^	75.78 ± 1.44^‡#^
Age (years)	43.95 ± 2.66	49.14 ± 3.42	44.07 ± 3.39
BMI (kg/m^2^)	24.40 ± 0.73	29.13 ± 1.02^‡^	28.21 ± 0.73^†^
WC (cm)	0.86 ± 0.02	0.92 ± 0.02^†^	0.89 ± 0.02

*P < .05 × NTs; ^†^ *P* < .01 × NTs, ^‡^ *P* < .001 × NTs, ^§^ *P* < .05 × HTs, ^#^ *P* < .001 × HTs.
